# An Equity Audit of a Statewide Cardiometabolic Risk Reduction Pilot Programme for Women with a History of Gestational Diabetes

**DOI:** 10.3390/nu18030489

**Published:** 2026-02-02

**Authors:** Yuqi Dou, Jacqueline A. Boyle, Jenna Van Der Velden, Jane Kwon, Carli Leishman, Elizabeth Holmes-Truscott, Kimberley L. Way, Timothy Skinner, Craig Pickett, Bei Bei, Siew Lim

**Affiliations:** 1 Health Systems and Equity, Eastern Health Clinical School, Monash University, Box Hill, Melbourne, VIC 3128, Australia; yuqi.dou@monash.edu (Y.D.); jacqueline.boyle@monash.edu (J.A.B.); 2 Diabetes Victoria, Carlton, Melbourne, VIC 3053, Australia; jvelden@diabetesvic.org.au (J.V.D.V.); jkwon@diabetesvic.org.au (J.K.); cleishman@diabetesvic.org.au (C.L.); 3 School of Psychology and Institute for Health Transformation, Deakin University, Geelong, VIC 3220, Australia; elizabeth.h@acbrd.org.au (E.H.-T.); t.skinner@deakin.edu.au (T.S.); 4 The Australian Centre for Behavioural Research in Diabetes, Diabetes Victoria, Carlton, Melbourne, VIC 3053, Australia; 5 Institute for Physical Activity and Nutrition, School of Exercise and Nutrition Sciences, Deakin University, Geelong, VIC 3220, Australia; kim.way@deakin.edu.au; 6 Exercise Physiology and Cardiovascular Health Lab, Division of Cardiac Prevention and Rehabilitation, University of Ottawa Heart Institute, Ottawa, ON K1Y 4W7, Canada; 7 Baker Heart and Diabetes Institute, Melbourne, VIC 3004, Australia; 8 Centre for Epidemiology and Statistics, Melbourne School of Population and Global Health, University of Melbourne, Carlton, Melbourne, VIC 3053, Australia; 9 Turner Institute for Brain and Mental Health, School of Psychological Sciences, Monash University, Melbourne, VIC 3800, Australia; bei.bei@monash.edu

**Keywords:** gestational diabetes mellitus, type 2 diabetes, engagement, completion, equity, migrants

## Abstract

Background: This equity audit assessed enrolment and completion of a state-funded cardiometabolic risk-reduction programme for women with prior gestational diabetes in Victoria, Australia. The analyses compared completion rates between the standard prevention programme *Life!* with one specifically adapted for women with prior gestational diabetes (*Life!* GDM) using the PROGRESS equity framework. Methods: Women with a history of GDM in the *Life!* GDM or the mainstream *Life!* programme in 2022–2025 were included. Multinomial logistic regression was used to impute categorical variables, logistic regression for binary variables, and linear regression for continuous variables. Estimates were combined across imputed datasets using Rubin’s rules. Results: A total of 2261 women were included: 370 in *Life!* GDM, and 1891 in *Life!* from 2022 to 2025, with completion rates of 36.7% and 52.2%, respectively. Compared with women in *Life!*, women in *Life!* GDM were more likely to come from non-English-speaking backgrounds, particularly South and Central Asian (30.5% vs. 17.0%) and South-East Asian backgrounds (13.0% vs. 4.3%). After multiple imputation, multivariable logistic regression showed that none of the examined participant characteristics were significantly associated with programme completion in *Life!* GDM. In the *Life!* cohort, completion was significantly associated with marital status, with single participants having lower odds of completion (OR = 0.59, 95% CI: 0.41–0.85), and with referral channel, with self-referral associated with higher odds of completion (OR = 1.71, 95% CI: 1.39–2.12). Conclusions: The adapted programme appeared to have reached more culturally and linguistically diverse women; however, lower completion among those experiencing disadvantage highlights the need for enhanced support and retention strategies to ensure equitable postpartum diabetes prevention.

## 1. Introduction

Gestational diabetes mellitus (GDM) is defined as glucose intolerance leading to hyperglycaemia with onset or first recognition during pregnancy [1,2]. Globally, GDM is estimated to affect nearly one in six live births, making it one of the most prevalent metabolic complications of pregnancy [3]. According to the National Hospital Morbidity Database, in 2021–2022, nearly one in five (18%) women aged 15–49 who gave birth in Australian hospitals were diagnosed with GDM [4]. After standardising for age over time, the incidence of GDM in Australia more than doubled between 2012–2013 and 2021–2022 [4]. This rapid increase is attributed to higher rates of maternal overweight and obesity, a growing proportion of the Australian population from ethnic backgrounds with a higher risk of GDM, advanced maternal age, and changes in diagnostic practices [4,5,6]. Beyond its immediate obstetric implications, GDM is also associated with substantial long-term health risks. These include elevated risk of GDM recurrence in subsequent pregnancies [7], type 2 diabetes (T2DM), and cardiovascular disease (CVD) [8]. For example, women with prior GDM have an estimated ten-fold higher risk of developing T2DM compared with women without a history of GDM [9]. This progression occurs disproportionately among those from socioeconomically disadvantaged, racial/ethnic minority, migrant, and culturally and linguistically diverse (CALD) backgrounds, highlighting persistent inequities across the reproductive life-course [10,11,12].

After GDM, clinical guidelines recommend ongoing T2DM screening (6–12 weeks postpartum and at least every three years thereafter), alongside advice and support to adopt health-promoting behaviours to reduce both T2DM and cardiovascular risk [7,13,14,15,16,17,18]. Long-term evidence from landmark behaviour change trials, such as the US Diabetes Prevention Program, the Finnish Diabetes Prevention Study, and the Da Qing Study, has established that a healthy diet (i.e., lower-fat, higher-complex-carbohydrate, higher fibre diet), increased physical activity, and weight loss can markedly reduce risk of T2DM [19,20,21,22,23,24,25,26]. Diabetes prevention programmes have also demonstrated effectiveness after GDM, with the adoption of health behaviours alone sufficient to reduce the risk of developing T2DM among women with prior GDM by around 17% [7,13,14,15,16,27]. However, postpartum mothers constitute a distinct population who encounter significant difficulties in sustaining healthy eating patterns and consistent participation in physical activity [28]. Low awareness of ongoing health risks, childcare responsibilities, and limited resources (including time, energy, information, and social support) can restrict women with a history of GDM from sustaining healthy diet and physical activity patterns or engaging with structured risk reduction programmes after childbirth [29]. Further, women from disadvantaged or minority backgrounds are particularly less likely to enrol in and more likely to disengage from such programmes, potentially exacerbating existing health disparities [30,31,32,33,34].

The *Life!* programme, funded by the Victorian state government and operating since 2007, is one of Australia’s longest-standing community-based diabetes prevention initiatives. The overarching aim of the programmes is to reduce the risk of T2DM and related cardiometabolic conditions. *Life!* is implemented across Victoria, Australia, and is also available to participants residing in selected eligible non-Victorian postcodes. In response to the growing prevalence of GDM and the unique needs of this cohort, a dedicated *Life!* GDM programme was introduced in 2022 to support women with a history of GDM in reducing their future cardiometabolic risk [35].

Despite the extensive evidence base on the efficacy and effectiveness of diabetes prevention programmes, population uptake is very low at less than 5% [36,37], and engagement varies across population groups [32]. Institutional and system-level factors, such as culturally inappropriate education and resources, may limit equitable access to the programmes and contribute to the under-representation of some groups in these programmes [3]. Health equity can be achieved when unfair and avoidable differences in health arising from social and structural conditions are removed, allowing all people to reach their full health potential [38]. Therefore, equitable access as measured in programme reach and completion are important implementation outcomes in nutrition and physical activity programmes aimed at reducing diabetes risks in at-risk populations. The PROGRESS framework is a useful framework to assess equity. The components of equity assessed in this framework include the following: Place of residence, Race/ethnicity/culture/language, Occupation, Gender/sex, Religion, Education, Socioeconomic status, and Social capital [39].

The aim of this study was to assess whether programme reach and completion varied across population and program subgroups, between the *Life!* GDM and the mainstream *Life!* programmes applying the PROGRESS framework.

## 2. Materials and Methods

### 2.1. Study Design and Participants

A retrospective cohort study was conducted using routinely collected data from the *Life!* programme. Participants provided consent at the point of the initial health check, during which they agreed to be contacted by Diabetes Victoria staff regarding the *Life!* program and for their personal information to be used for essential programme administrative purposes. This study was approved as an amendment to the Chinese and South Asian Vanguard ethics application (ID 44150). Participant data were obtained at two time points: at the introductory session (Time 1, baseline) and at Session five (Time 2, 5–6 months, completion).

Eligibility for enrolment into the *Life!* programme requires at least one recognised cardiometabolic risk factor. Participants were eligible if they recorded an AUSDRISK score ≥12 together with a BMI ≥ 25 kg/m^2^ (or ≥23 kg/m^2^ for those self-identifying as Asian, including the Indian sub-continent), had an intermediate or high CVD risk score, or had a pre-existing condition associated with increased risk of CVD or T2DM, including a previous diagnosis of GDM [40]. For this analysis, we extracted records of women residing in Victoria who had documented prior GDM, enrolling between 1 July 2018 and 25 February 2025.

Eligibility for enrolment into *Life!* GDM was restricted to women with a previous history of GDM (at least 6 weeks after birth) who were living in Victoria, able to communicate in English, and under 50 years of age. Participants were also required not to be currently pregnant or diagnosed with diabetes at the time of enrolment. For this analysis, we extracted records of all women enrolling from the programme’s commencement on 1 July 2022 through to 25 February 2025.

### 2.2. Description of the Life! and the Life! GDM Programmes

The *Life!* programme consists of a one-on-one introductory session, followed by six structured group-based education sessions over 12 months (or one-on-one telephone health coaching) [41]. Group sessions are delivered online or face-to-face in English (mainstream), as well as culturally and linguistically diverse (CALD) program sub-types, including Chinese, Vietnamese, and Arabic, while telephone health coaching is provided in both English and Chinese. During the COVID-19 pandemic period, all *Life!* programme sessions were delivered online. Across both group-based and telephone health coaching programmes, participants receive structured education and behavioural coaching focused on healthy eating (reducing total and saturated fat intake, reducing total energy intake, increasing fibre intake), regular physical activity, effective weight management, and strategies to support sustainable behavioural change. Core components address dietary patterns, energy balance, portion control, physical activity, stress and sleep management, overcoming behavioural barriers, relapse prevention, and the development of sustainable health behaviour strategies [41]. Participants complete modified SMART goal setting to emphasise monitorable progress and action-oriented behaviours, supporting tracking and engagement. More programme details can be found in Figure S1. For the mainstream *Life!* programme, recruitment pathways involved a combination of paid advertising campaigns and community engagement strategies. Promotional materials (flyers, brochures, and posters) were distributed in primary care settings, community health services, and other relevant locations. Recruitment was further supported by a dedicated programme website and direct engagement with healthcare providers, primarily general practices and community health organisations.

Developed in partnership with women with a history of GDM, facilitators, health professionals, developers, and community stakeholders, *Life!* GDM is delivered exclusively online, allowing participants to join from the comfort of their homes to meet the specific health needs of postpartum women who recently had GDM [35]. Key adaptations include clearer health messaging on cardiometabolic risks after GDM and the addition of information on stress, sleep, and breastfeeding, acknowledging their impact on diet, physical activity, and overall well-being. Weight-loss goals are based on pre-pregnancy weight, with a focus on achieving a return to pre-pregnancy levels or a 5% reduction in weight for those with a BMI over 25 kg/m^2^. Physical activity recommendations were adapted to consider postpartum pelvic floor health, with a gradual start focusing on pelvic floor exercises and the inclusion of at least two days of resistance training each week to support both physical and mental health. The programme was also revised to include more relatable (i.e., of women with past GDM) and successful examples of behaviour change to help participants set realistic and achievable goals. Women were recruited through multiple channels, including promotional materials (flyers, brochures, and posters) distributed across the GDM care continuum and a dedicated landing page on the *Life!* GDM website. Recruitment also involved two statewide National Diabetes Services Scheme (NDSS), central email campaigns sent to 47,948 Victorian women aged between 18 and 49 years with a history of GDM, as well as engagement with healthcare providers. This included outreach to 438 General Practices and 270 Maternal and Child Health/Community Health Services and private obstetric clinics, which were provided with programme information to support referrals.

### 2.3. Outcomes and Explanatory Variables

The primary outcome of this audit was programme completion (the attendance at Session five). Explanatory variables were selected based on the PROGRESS-Plus framework for equity [39]. Place of residence (P) was classified as either metropolitan Melbourne or regional Victoria, representing areas within and outside the Melbourne metropolitan boundary, respectively. The Race/Ethnicity/Culture/Language (R) was evaluated using participants’ country of birth and cultural background as proxy measures for race, ethnicity, and culture. Following the Australian Bureau of Statistics Standard Classification of Cultural and Ethnic Groups, they were categorised into nine cultural regions [42]. Language background was coded as “yes” or “no” to indicate whether participants self-identified as having an English-speaking background. Occupation (O) was self-reported and categorised as employed, unemployed, retired, home duties, or student. Educational status (E) was categorised based on whether participants had completed any tertiary-level qualification. Socioeconomic status (SES) (S) was based on participants’ self-reported annual pre-tax household income, which was grouped into low-, medium-, or high-income categories. Detailed classification is provided in Table S1.

Apart from these PROGRESS equity variables, additional variables were also included to account for individual and programme-related factors potentially influencing programme completion. Current age was recorded as a continuous variable at enrolment. Marital status was categorised as de facto/married, divorced/separated, or never married/widowed. Smoking status was self-reported and categorised as yes or no. Referral channel referred to the pathway through which participants entered the programme and was classified as health facilitator/provider, health professional/GP/pharmacist, self-referral, or unknown.

### 2.4. Statistical Analysis

All statistical analyses were performed using R software (version 4.2.2). To maximise comparability, our cohort definition was intentionally structured around calendar time as *Life!* GDM was launched in 2022. We also have data from a historical cohort (*Life!* 2018–2021) for comparison; see the Supplementary Materials for details. Participants were classified into two groups according to programme type and period to reflect differences in programme delivery and availability: *Life!* GDM (2022–2025) and *Life!* (2022–2025). The data for this analysis only includes the *Life!* mainstream programme in English, as *Life!* GDM is delivered exclusively in English. In addition, for *Life!,* we included only participants who attended group sessions, as *Life!* GDM is delivered exclusively in a group-based format and not through telephone health coaching (other stream). Restricting the comparison group to English-language participants and a group-based format ensures consistency in comparison between programme deliveries and reduces potential confounding related to language.

Descriptive statistics were used to summarise participants’ sociodemographic characteristics. Continuous variables were presented as median and interquartile range (IQR). Categorical variables, including income level, marital status, smoking status, country of birth, cultural background, English-speaking background, referral channel, education level, employment status, and area of residence, were expressed as frequencies and proportions [n (%)], with missing values explicitly retained as a “Unknown” category. Between-group differences in distribution were quantified using the absolute percentage-point difference (% difference) for categorical variables. To assess the magnitude of imbalance independent of sample size, standardised differences were calculated for each variable and reported as the standardised mean difference (SMD): for continuous variables, the difference in means divided by the pooled standard deviation; for categorical variables, standardised differences were derived from group-specific proportions, summarised as an overall measure for each variable.

Logistic regression models were fitted to identify factors associated with programme completion (dependent variable: 1 = completed, 0 = not completed). Independent variables included sociodemographic characteristics (area of residence, country of birth, English-speaking background, education, employment, income, and marital status), age, smoking status, and referral channel. Both univariable and multivariable logistic regression models were fitted, with the latter presented as a complete-case analysis. Subsequently, due to the risk of selection bias and with the assumption of data missing completely at random (MCAR) with the complete-case analysis, multiple imputation was performed. Missing data in independent variables were handled using multiple imputation by chained equations under a missing-at-random assumption (MAR) [43]. All variables included in the analysis, including the outcome, were incorporated in the imputation models. Multinomial logistic regression was used to impute categorical variables, logistic regression for binary variables, and linear regression for continuous variables. Estimates were combined across imputed datasets using Rubin’s rules [43].

Multicollinearity among independent variables was assessed using the variance inflation factor (VIF), with variables exhibiting a VIF ≥ 5 excluded from the final model. A two-sided *p*-value < 0.05 was considered statistically significant. Model calibration was assessed using the Hosmer–Lemeshow goodness-of-fit test, with *p*-value > 0.05 indicating adequate model fit.

## 3. Results

### 3.1. Comparison Between Life! GDM and Life! Participants

Table 1 summarises the baseline characteristics of the *Life!* GDM and *Life!* participants. Participants in *Life!* GDM were more likely to reside in metropolitan areas than those in *Life!* 2022–2025 (85.9% vs. 79.0%; % difference = 7.0; SMD = 0.185). Education level followed a similar pattern (% difference = 26.4; SMD = 0.693), with tertiary education reported by 92.7% of the *Life!* GDM cohort compared with 66.3% in *Life!* groups. High-income status was also more common in *Life!* GDM (27.3% vs. 5.4%), as were home duties (18.9% vs. 8.8%). Country of birth differed markedly between groups (% difference = 23.7; SMD = 0.488): a greater proportion of participants in *Life!* were born in Oceania (62.1% vs. 38.4% in *Life!* GDM), whereas *Life!* GDM had higher proportions of participants from South and Central Asia (30.5% vs. 17.0%) and South-East Asia (13.0% vs. 4.3%). Aligned with this, English-speaking backgrounds were more common in *Life!* than in *Life!* GDM (65.8% vs. 41.4%; % difference = 24.5; SMD = 0.507). Referral channels also differed substantially (% difference = 33.5; SMD = 0.917), with self-referral far more common in *Life!* GDM than the *Life!* programme 2022–2025 (96.5% vs. 63.0%).

### 3.2. Programme Completion by Participant Characteristics

Tables S2–S4 summarise the completion status across all cohorts, showing that 36.7% of *Life!* GDM participants completed the programme, compared with 52.2% of those enrolled in the *Life!* programme between 2022 and 2025 and 57.7% among those enrolled in 2018–2021.

#### 3.2.1. Programme Completion by Participant Characteristics in *Life!* GDM

The results of multivariable logistic regression after multiple imputation (Table 2) indicated that none of the examined participant characteristics were significantly associated with programme completion in *Life!* GDM. Univariable regression results and complete-case analyses are presented in Tables S7 and S9, respectively. Complete-case analyses were highly consistent with the multiple imputation results; however, marital status remained significant, with divorced/separated participants having lower odds of completion (AOR = 0.06, 95% CI: 0.01–0.47).

#### 3.2.2. Programme Completion by Participant Characteristics in *Life!* (2022–2025)

Regression analyses of programme completion by participant characteristics in *Life!* (2022–2025) after multiple imputation are presented in Table 3. Marital status was significantly associated with completion, with single participants having lower odds of completion (OR = 0.59, 95% CI: 0.41–0.85, *p* = 0.005). Referral channel was also significant: participants who self-referred had higher odds of completion (OR = 1.71, 95% CI: 1.39–2.12, *p* < 0.001). Univariable regression results and complete-case analyses are presented in Tables S8 and S10, respectively. The complete-case results were broadly consistent with the multiple imputation analyses, except that employment status (home duties) was significant (AOR = 0.61, 95% CI: 0.41–0.92, *p* = 0.017), while referral channel was no longer statistically significant.

### 3.3. Synthesis of Findings: Disparities, Strategies, and Evidence Gaps

To facilitate rapid interpretation of the findings and their implications, we present two summary tables. Table 4 synthesises the key dimensions of disparities in programme completion and maps these to potential policy and programme strategies. Table 5 complements this by outlining the principal evidence and data gaps identified in the equity audit and highlighting priorities for future research and routine data collection to strengthen equity-focused evaluation and service improvement.

## 4. Discussion

To determine the equitable reach of *Life!* GDM, this audit compared the sociodemographic characteristics of women with a history of GDM enrolled into the *Life!* GDM and mainstream *Life!* programmes and explored factors associated with programme completion within each cohort. Women enrolled in *Life!* GDM were more likely to be from non-English-speaking backgrounds, particularly from South and Central Asian or South-East Asian backgrounds. They were also more likely to reside in metropolitan areas, have higher household incomes, hold tertiary qualifications, be in de facto or married relationships, and self-refer into the programme. In contrast, participants in the *Life!* programme were relatively more likely to be of Oceanian background, reside in regional areas, have no tertiary education, come from lower-income households, and enter the programme through professional referral.

The *Life!* GDM appeared to have reached more women from South and Central Asian and South-East Asian backgrounds than the *Life!* programme. In Australia, ethnicity is a recognised risk factor for GDM, with higher prevalence reported among women of Asian (including the Indian sub-continent), Aboriginal, Torres Strait Islander, Pacific Islander, Māori, Middle Eastern, and non-white African descent [4]. Given that women of Asian descent are more likely to develop T2DM than those of European ancestry, their higher enrolment in *Life!* GDM likely mirrors their elevated physiological risk [9,44,45,46,47]. Greater reach of these populations in *Life!* GDM represent progression towards equitable health outcomes given their higher underlying susceptibility to GDM. However, it should be noted that the analysis of *Life!* data in the current study comprised participants in the mainstream English-language programme only. Other language sub-types were not included in this analysis, which may have influenced the distribution of country of birth and cultural background. It should also be noted that despite the higher number of South and Central Asian and South-East Asian women in the *Life!* GDM programme, the true response rate, defined as the percentage of those eligible reached by this programme at a state population level, remains unknown. Our past research indicates high proportions of eligible participants remain unreached [42], suggesting more work is needed to improve the reach of cardiometabolic risk reduction programmes [37].

The strong association between self-referral and programme completion is consistent with evidence from self-determination theory and behaviour change intervention studies, which show that autonomous motivation and self-regulated decision-making are important predictors of long-term engagement [48]. Conversely, passive-driven referrals may fail to generate the level of personal commitment needed to maintain long-term enrolment [49]. Apart from greater motivation or health literacy, self-referral may also be shaped by how information about the programme was distributed and the recruitment pathways through which women first encountered it (e.g., the postpartum life stage at which information was received and the nature of the contacts providing that information). As these potential mechanisms are not captured in routinely collected programme data, we cannot determine why self-referral is associated with higher completion in this audit, and this could be an important aspect to explore further in future.

The significant association between marital status and completion indicates that family support and time availability may play a key role in sustaining engagement in postpartum behaviour change programmes. Time–poverty theory suggests that single-parent households experience a disproportionate burden of competing demands, as the responsibilities of earning an income and providing childcare fall on one individual [50,51]. In contrast, partnered mothers may benefit from shared labour across paid work and household duties [50]. Apart from time availability, household partners often share lifestyle behaviours and health risks, and if they also value positive health behaviours, can be enablers to supporting women’s health in the postpartum period [52,53] through their accompaniment, encouragement, and reminders in sustaining healthy behaviours [53,54,55,56,57]. In a cardiovascular lifestyle intervention, having a partner was associated with a higher likelihood of successful lifestyle risk-factor modification (aRR 1.93, 95% CI 1.40–2.51), and partner participation was associated with successful weight reduction (aRR 1.73, 95% CI 1.15–2.35) [53]. However, the exact mechanisms of how marital status affects postpartum women’s engagement in health behaviour have not been investigated.

Qualitative and survey evidence from women with prior GDM consistently shows that childcare responsibilities, infant care, home duties, and a lack of family support are major barriers to engaging in and sustaining diabetes risk-reduction behaviours, often leading to non-participation or early disengagement [58,59,60]. In many households, stay-at-home mothers are socially expected to assume the primary role in managing domestic and caregiving responsibilities [61]. These expectations often reduce the extent to which other family members share household tasks, leaving mothers with limited practical support [61]. Such constrained support can intensify time pressures and may hinder their ability to engage consistently in self-care, including healthy behaviours such as healthy eating and exercise. Given that the *Life!* GDM programme specifically targets the postpartum period when childcare demands are highest, these barriers may be even more pronounced. In our audit, women whose main role was home duties were less likely to complete the programme, consistent with literature indicating that competing demands within the home environment, coupled with limited family support, may influence sustained participation. The relationship between employment and health in women requires further research, particularly in the context of engaging in healthy eating and exercise during the postpartum period.

### Strengths and Limitations

A key strength of this study is the application of the PROGRESS framework to conduct a structured equity audit of a statewide cardiometabolic prevention programme, enabling systematic examination of how social and structural determinants shape enrolment and completion patterns. By integrating routinely collected programme data from multiple sites, this study provides a real-world evaluation of programme engagement and completion among women with a history of GDM.

Several limitations should also be acknowledged. First, as a retrospective audit of administrative data, the analysis was restricted to routinely collected quantitative variables, which may not include variables along the complex pathways linking social determinants to health behaviour. Other methodologies, such as qualitative or mixed-method research on lived experience, may be needed for further exploration. In addition, reasons for non-completion were not systematically recorded, limiting interpretation of disengagement. Second, response rates were not available, limiting the ability to evaluate programme reach. Third, this analysis included only the English-language mainstream *Life!* programme and (mostly online) group sessions to allow comparability to the *Life!* GDM programme. As a result, inequities experienced by culturally and linguistically diverse participants and those relying on alternative delivery models (e.g., telephone health coaching) could not be determined. Fourth, as this was an observational study, our findings describe associations rather than causal effects. The use of routinely collected data also meant that residual confounding from unmeasured factors may remain. Finally, missing data for key variables (e.g., income, education, marital status) was partly driven by privacy-related “prefer not to respond” options; therefore, residual bias may remain even after imputation, and findings should be interpreted cautiously.

In the present audit, our primary contribution is to identify priority populations experiencing lower completion within existing service models. However, addressing inequities in postpartum diabetes prevention at scale will likely require broader, multi-level responses beyond a single programme targeting individual behaviour change. Future approaches could combine diabetes prevention programmes with other policy and community-wide strategies (e.g., childcare access, flexible work conditions, and strengthened social support). These changes are outside the remit of the *Life!* programme but are critical considerations for reducing inequities in GDM-related outcomes.

## 5. Conclusions

This statewide audit evaluated a community-based nutrition and physical activity programme for diabetes risk reduction aimed at women with a history of GDM in Victoria, Australia. *Life!* GDM appeared to have reached more migrant women, such as South and Central Asian and South-East Asian, than the *Life!* programme, suggesting more effective reach pathways in this programme for these priority groups. However, lower completion rates indicate persistent barriers to sustained engagement in nutrition and physical activity changes among those experiencing disadvantage. These findings highlight the need to strengthen strategies that enhance both reach and retention in diabetes prevention programmes comprising nutrition and physical activity, particularly among women facing social disadvantages.

## Figures and Tables

**Table 1:** Baseline characteristics of the *Life!* GDM and *Life!* participants with a history of GDM during 2022–2025.

Variable		*Life!* GDM (N = 370)	*Life!* (N = 1891)	% Difference	SMD
P	Area			7.0	0.185
	Metropolitan	318 (85.9)	1493 (79.0)		
	Regional	52 (14.1)	398 (21.0)		
R	Country of Birth			23.7	0.488
	Oceania	142 (38.4)	1174 (62.1)		
	North-West Europe	12 (3.2)	58 (3.1)		
	South and East Europe	4 (1.1)	41 (2.2)		
	North Africa and Middle East	12 (3.2)	44 (2.3)		
	South-East Asia	48 (13.0)	81 (4.3)		
	North-East Asia	20 (5.4)	83 (4.4)		
	South and Central Asia	113 (30.5)	322 (17.0)		
	America (North and South)	8 (2.2)	39 (2.1)		
	Sub-Saharan Africa	11 (3.0)	37 (2.0)		
	Unknown	0 (0.0)	12 (0.6)		
	Cultural Background			23.6	0.486
	Oceania	141 (38.1)	1167 (61.7)		
	North-West Europe	12 (3.2)	56 (3.0)		
	South and East Europe	4 (1.1)	54 (2.9)		
	North Africa and Middle East	10 (2.7)	43 (2.3)		
	South-East Asia	46 (12.4)	73 (3.9)		
	North-East Asia	24 (6.5)	88 (4.7)		
	South and Central Asia	117 (31.6)	318 (16.8)		
	America (North and South)	6 (1.6)	38 (2.0)		
	Sub-Saharan Africa	9 (2.4)	32 (1.7)		
	Unknown	1 (0.3)	22 (1.2)		
	English-Speaking Background			24.5	0.507
	Yes	153 (41.4)	1245 (65.8)		
	No	217 (58.6)	646 (34.2)		
O	Employment Status			14.8	0.490
	Employed	264 (71.4)	1275 (67.4)		
	Home duties	70 (18.9)	167 (8.8)		
	Unemployed/Retired	14 (3.8)	88 (4.7)		
	Student	9 (2.4)	14 (0.7)		
	Unknown	13 (3.5)	347 (18.4)		
E	Education Level			26.4	0.693
	Tertiary education	343 (92.7)	1253 (66.3)		
	No tertiary education	17 (4.6)	215 (11.4)		
	Unknown	10 (2.7)	423 (22.4)		
S	Income Level			21.9	0.620
	High	101 (27.3)	102 (5.4)		
	Medium	155 (41.9)	750 (39.7)		
	Low	51 (13.8)	500 (26.4)		
	Unknown	63 (17.0)	539 (28.5)		
Others	Current Age at Enrolment				−0.266
		38.0 (35.0, 41.0)	37.0 (34.0, 43.0)		
	Marital Status			17.8	0.591
	De facto/married	337 (91.1)	1385 (73.2)		
	Divorced/separated	14 (3.8)	81 (4.3)		
	Never married/widowed	12 (3.2)	59 (3.1)		
	Unknown	7 (1.9)	366 (19.4)		
	Do you Smoke Daily?			0.6	0.044
	No	363 (98.1)	1843 (97.5)		
	Yes	7 (1.9)	47 (2.5)		
	Unknown	0 (0.0)	1 (0.1)		
	Referral Channel			33.5	0.917
	Health Facilitator/Provider	1 (0.3)	492 (26.0)		
	Health Professional/GP/Pharmacist	8 (2.2)	208 (11.0)		
	Self	357 (96.5)	1191 (63.0)		
	Unknown	4 (1.1)	0 (0.0)		

SMD, standardised mean difference; GDM, gestational diabetes mellitus; GP, general practitioner.

**Table 2:** Regression analyses of programme completion by participant characteristics in *Life!* GDM (2022–2025) after multiple imputation.

Variable	Category	OR	Std. Err.	*t*	*p*	95% CI
Area	Metropolitan (ref)					
	Regional	0.98	0.33	−0.05	0.957	0.51–1.91
Country of birth	Oceania (ref)					
	Europe and Americas	1.09	0.68	0.15	0.885	0.33–3.67
	Africa and Middle East	1.24	1.00	0.27	0.789	0.26–5.98
	East Asia	1.61	1.26	0.61	0.540	0.35–7.43
	South and Central Asia	0.83	0.64	−0.24	0.808	0.19–3.72
English-speaking background	Yes (ref)					
	No	0.62	0.45	−0.66	0.509	0.15–2.54
Education level	Tertiary education (ref)					
	Not tertiary	0.86	0.52	−0.24	0.809	0.26–2.82
Employment status	Employed (ref)					
	Home duties	1.28	0.41	0.78	0.438	0.69–2.39
	Retired/Unemployed	1.59	1.01	0.73	0.465	0.46–5.53
	Student	0.36	0.39	−0.95	0.342	0.04–2.98
Income level	High (ref)					
	Moderate	0.89	0.24	−0.43	0.668	0.52–1.52
	Low	0.63	0.27	−1.09	0.275	0.27–1.45
Marital status	De facto/married (ref)					
	Single (divorced, separated, widowed, or never married)	0.37	0.20	−1.84	0.066	0.13–1.07
Current age	(continuous)	1.02	0.02	0.89	0.374	0.98–1.07
Smoker	No (ref)					
	Yes	0.62	0.54	−0.55	0.584	0.11–3.45
Referral channel	Healthcare professional (ref)					
	Self	0.71	0.55	−0.44	0.659	0.16–3.23
Constant		0.54	0.64	−0.52	0.602	0.05–5.57

GDM, gestational diabetes mellitus; OR, odds ratio.

**Table 3:** Regression analyses of programme completion by participant characteristics in *Life!* (2022–2025) after multiple imputation.

Variable	Category	OR	Std. Err.	*t*	*p*	95% CI
Area	Metropolitan (ref)					
	Regional	1.03	0.12	0.28	0.780	0.82–1.31
Country of birth	Oceania (ref)					
	Europe and Americas	1.28	0.33	0.96	0.336	0.78–2.11
	Africa and Middle East	0.70	0.23	−1.10	0.270	0.37–1.32
	East Asia	1.20	0.40	0.55	0.582	0.62–2.31
	South and Central Asia	0.93	0.29	−0.24	0.811	0.50–1.72
English-speaking background	Yes (ref)					
	No	0.77	0.22	−0.93	0.352	0.44–1.34
Education level	Tertiary education (ref)					
	Not tertiary	1.26	0.20	1.49	0.138	0.93–1.72
Employment status	Employed (ref)					
	Home duties	0.83	0.15	−1.04	0.300	0.58–1.19
	Retired/Unemployed	0.88	0.23	−0.50	0.619	0.52–1.47
	Student	1.17	0.69	0.26	0.797	0.36–3.78
Income level	High (ref)					
	Moderate	1.23	0.28	0.91	0.366	0.79–1.92
	Low	1.15	0.28	0.60	0.553	0.71–1.87
Marital status	De facto/married (ref)					
	Single (divorced, separated, widowed, or never married)	0.59	0.11	−2.82	**0.0** **05**	0.41–0.85
Current age	(continuous)	1.01	0.01	1.60	0.109	1.00–1.02
Smoker	No (ref)					
	Yes	0.82	0.25	−0.66	0.507	0.45–1.49
Referral channel	Healthcare professional (ref)					
	Self	1.71	0.18	5.01	**<0.0** **0** **1**	1.39–2.12
Constant		0.52	0.17	−2.01	0.045	0.28–0.99

GDM, gestational diabetes mellitus; OR, odds ratio.

**Table 4:** Key dimensions of disparities in programme completion and potential policy and programme strategies.

Key Dimensions of These Disparities in Programme Completion	Potential Policy and Programme Strategies
Programme type: Completion was lower in *Life!* GDM (36.7%) than in the contemporaneous mainstream *Life!* programme (52.2%) during 2022–2025.	Strengthen retention in *Life!* GDM: proactive follow-up after enrolment (welcome call/SMS), early screening for barriers to engagement, and stepped-up support for those at risk of dropout. Increase flexibility to fit postpartum schedules: shorter modules, evening/weekend options, pause–resume pathways, and telephone-first delivery where needed. Understand participant perspectives: incorporate brief post-enrolment and post-dropout feedback (e.g., short surveys or interviews) to capture women’s views on programme relevance, burden, and acceptability, and use these insights to refine programme design and delivery.
Engagement differences clustered around language/cultural background, socioeconomic resources, caregiving demands (home duties), metropolitan access, and referral pathway (self-referral).	➢ Strengthen multilingual engagement and follow-up, expand proactive referrals beyond self-referral, and offer flexible, childcare-friendly options informed by participant feedback.
Marital status: Single women had lower odds of programme completion than de facto/married women (observed in adjusted models).	Avoid assuming household/partner support in promotional materials. Strengthen linkage to social and community supports (e.g., parenting, psychosocial, and practical support services). Understand the barriers faced by single or unmarried women in the programme in further research.
Employment status: Women reporting home duties had lower odds of completion than employed women (observed in the mainstream *Life!* cohort)	➢ Increase flexibility to reduce time burden (evening/weekend options, and shorter sessions). ➢ Provide proactive retention supports (reminders, brief check-ins, tailored scheduling around caregiving demands). ➢ Deliver micro-learning modules (e.g., short videos and small, actionable tasks) that participants can complete in brief intervals to fit around caregiving and time constraints. ➢ Understand the barriers faced by women not in paid employment in future research.
Referral pathway: Completion differed by referral channel, with a trend toward higher completion among self-referred participants in the mainstream *Life!* cohort.	Strengthen clinician-initiated referral through standardised prompts in postpartum care pathways (GP/maternity/child health). Send letters of invitation through the NDSS platform. Provide plain-language explanation of benefits, time commitment, and what “completion” means; emphasise postpartum relevance for prior GDM.
Differences by country of birth and English-speaking background were evident in crude analyses but were not consistently retained after adjustment.	➢ Improve cultural and linguistic accessibility (multilingual/low-literacy materials, interpreter/bilingual facilitation where feasible). ➢ Provide cultural adaptation engagement strategies (CALD stream, and cultural lifestyle adaptation).

**Table 5:** Key evidence and data gaps identified in the equity audit, and priorities for future research and routine data collection.

Evidence/Data Gap	Why It Matters for Equity, Reach, and Completion
Health literacy	Limits ability to untangle whether higher completion (e.g., self-referral) reflects programme factors vs. participant motivation
Motivation	
Time since GDM diagnosis	Engagement barriers and perceived risk vary by postpartum stage; type 2 diabetes risk varies by postpartum age; limits subgroup analyses and targeting
Reasons for dropout	Limits identification of key causes of attrition; unable to develop tailored support to address retention
Social capital/community resources (PROGRESS: Social capital)	Key equity dimension missing; limits PROGRESS coverage
Baseline cardiometabolic risk profile (BMI, HbA1c, Blood Pressure, lipids)	Limits assessment of whether reach/completion differs by underlying risk and need
Recruitment/marketing exposure	Self-referral may reflect distribution/recruitment pathways rather than individual readiness alone; mechanisms cannot be disentangled

## Data Availability

The datasets presented in this article are not readily available, as data sharing may compromise participant anonymity. Requests to access the datasets should be directed to the corresponding author.

## Supplementary Materials

The following supporting information can be downloaded at: https://www.mdpi.com/article/10.3390/nu18030489/s1, Table S1: Income classification; Table S2: Baseline characteristics of the *Life!* GDM (2022–2025), *Life!* (2022–2025) and *Life!* (2018–2021) participants with a history of GDM; Table S3: Programme Completion by participant characteristics in *Life!* GDM (2022–2025); Table S4: Programme Completion by participant characteristics in *Life!* (2022–2025); Table S5: Programme Completion by participant characteristics in *Life!* (2018–2021); Table S6: Regression analyses of programme completion by participant characteristics in *Life!* participants (2018–2021); Table S7: Univariable regression analyses of programme completion by participant characteristics in *Life!* GDM (2022–2025); Table S8: Univariable regression analyses of programme completion by participant characteristics in *Life!* (2022–2025); Table S9: Multivariable regression analyses of programme completion by participant characteristics in *Life!* GDM participants with a history of GDM (2022–2025); Table S10: Multivariable regression analyses of programme completion by participant characteristics in *Life!* participants with a history of GDM (2022–2025); Figure S1: Programme design.

## Abbreviations

The following abbreviations are used in this manuscript:

GDM	Gestational Diabetes Mellitus
T2DM	Type 2 Diabetes Mellitus
CALD	Culturally and Linguistically Diverse Backgrounds
CVD	Cardiovascular Disease
SES	Socioeconomic Status
COR	Crude Odds Ratio
AOR	Adjusted Odds Ratio
CI	Confidence Interval
GP	General Practitioner
