# Supplementary material for: An Equity Audit of a Statewide Cardiometabolic Risk Reduction Pilot Programme for Women with a History of Gestational Diabetes
Source: Nutrients. 2026 Feb 2;18(3):489. doi: 10.3390/nu18030489 (PMC12899937; doi:10.3390/nu18030489)
Supplement: Supplementary file 1 [file nutrients-18-00489-s001.zip › nutrients-4075755-supplementary.pdf]

An Equity Audit of a Statewide Cardiometabolic Risk Reduction Pilot Program for Women with a History of Gestational Diabetes

Yuqi Dou [1], Jacqueline A. Boyle [1], Jenna Van Der Velden [2], Jane Kwon [2], Carli Leishman [2], Elizabeth Holmes-Truscott [3,4], Kimberley L. Way [5,6,7], Timothy Skinner [3,4], Craig Pickett [8], Bei Bei [9] and Siew Lim [1,*]

1 Health Systems and Equity, Eastern Health Clinical School, Monash University, Box Hill, Melbourne, VIC 3128, Australia; yuqi.dou@monash.edu (Y.D.); jacqueline.boyle@monash.edu (J.A.B.)
2 Diabetes Victoria, Carlton, Melbourne, VIC 3053, Australia; jvelden@diabetesvic.org.au (J.V.D.V.); jkwon@diabetesvic.org.au (J.K.); cleishman@diabetesvic.org.au (C.L.)
3 School of Psychology and Institute for Health Transformation, Deakin University, Geelong, VIC 3220, Australia; elizabeth.h@acbrd.org.au (E.H.-T.); t.skinner@deakin.edu.au (T.S.)
4 The Australian Centre for Behavioural Research in Diabetes, Diabetes Victoria, Carlton, Melbourne, VIC 3053, Australia
5 Institute for Physical Activity and Nutrition, School of Exercise and Nutrition Sciences, Deakin University, Geelong, VIC 3220, Australia; kim.way@deakin.edu.au
6 Exercise Physiology and Cardiovascular Health Lab, Division of Cardiac Prevention and Rehabilitation, University of Ottawa Heart Institute, Ottawa, ON K1Y 4W7, Canada
7 Baker Heart and Diabetes Institute, Melbourne, VIC 3004, Australia
8 Centre for Epidemiology and Statistics, Melbourne School of Population and Global Health, University of Melbourne, Carlton, Melbourne, VIC 3053, Australia
9 Turner Institute for Brain and Mental Health, School of Psychological Sciences, Monash University, Melbourne, VIC 3800, Australia; bei.bei@monash.edu
* Correspondence: siew.lim1@monash.edu

Outline:

Table S1. Income classification

Table S2. Baseline characteristics of the *Life!* GDM (2022-2025), *Life!* (2022-2025) and *Life!* (2018-2021) participants with a history of GDM

Table S3. Program completion by participant characteristics in *Life!* GDM (2022-2025)

Table S4. Program completion by participant characteristics in *Life!* (2022-2025)

Table S5. Program completion by participant characteristics in *Life!* (2018-2021)

Table S6. Regression analyses of program completion by participant characteristics in *Life!* participants (2018-2021)

Table S7. Univariable regression analyses of program completion by participant characteristics in *Life!* GDM (2022-2025).

Table S8. Univariable regression analyses of program completion by participant characteristics in *Life!* (2022-2025).

Table S9. Multivariable regression analyses of program completion by participant characteristics in *Life!* GDM participants with a history of GDM (2022-2025)

Table S10. Multivariable regression analyses of program completion by participant characteristics in *Life!* participants with a history of GDM (2022-2025)

Figure S1. Program design

Table S1. Income classification

Income Level Income	Income Range	Tick below
Low income range	• People receiving a full or part pension.<br>• People with a health care card.<br>• Single people with a before-tax income of under $39,089.<br>• Couples with a before-tax income of under $59,802.<br>• Families with one child with a before-tax income of under $66,009 (plus $6,206 for each extra dependent child).<br>• Any of the above who face additional costs (due to disability and/or other factors) which reduces their income to this level.	
Medium income range	• Single people with a before-tax income greater than $39,089 but less than $86,208.<br>• Couples with a before-tax income greater than $59,802 but less than $115,245.<br>• Families with one child with a before-tax income greater than $66,009 but less than $118,546 (plus $6,206 for each extra dependent child).<br>• Any of the above who face additional costs (due to disability and/or other factors) which reduces their income to this level.	
High income range	• Single people with a before-tax income of more than $86,208.<br>• Couples with a before-tax income of more than $115,245.<br>• Families with one child with a before-tax income of more than $118,546 (plus $6,206 for each extra dependent child).	

*Adapted from: Department of Health and Human Services Victorian Home and Community Care (HACC) Fees Policy. Summary of Fees and Income Ranges, current as at November 2025.

Table S2. Baseline characteristics of the *Life!* GDM (2022-2025), *Life!* (2022-2025) and *Life!* (2018-2021) participants with a history of GDM.

	Variable		*Life!* GDM (N=370) 2022-2025	*Life!* program (N=2645) 2022-2025 (n=1891)	2018-2021 (n=754)	*p*-value
**P**	**Area**	**Metropolitan**	318 (85.9)a, b	1493 (79.0)a, c	562 (74.5)b, c	**<0.001**
		**Regional**	52 (14.1)a, b	398 (21.0)a, c	192 (25.5)b, c	
**R**	**Country of Birth**	**Oceanian**	142 (38.4)a, b	1174 (62.1)a	486 (64.5)b	**<0.001**
		**North West Europe**	12 (3.2)	58 (3.1)	31 (4.1)	
		**South & East Europe**	4 (1.1)	41 (2.2)	25 (3.3)	
		**North Africa & Middle East**	12 (3.2)	44 (2.3)	18 (2.4)	
		**South East Asia**	48 (13.0)a, b	81 (4.3)a	31 (4.1)b	
		**North East Asia**	20 (5.4)b	83 (4.4)c	13 (1.7)b, c	
		**South and Central Asia**	113 (30.5)a, b	322 (17.0)a	115 (15.3)b	
		**America (North & South)**	8 (2.2)	39 (2.1)	15 (2.0)	
		**Sub Saharan Africa**	11 (3.0)	37 (2.0)	14 (1.9)	
		**Unknown**	0 (0.0)	12 (0.6)	6 (0.8)	
	**Cultural Background**	**Oceanian**	141 (38.1)a, b	1167 (61.7)a, c	419 (55.6)b, c	**<0.001**
		**North West Europe**	12 (3.2)	56 (3.0)	31 (4.1)	
		**South & East Europe**	4 (1.1)b	54 (2.9)	31 (4.1)b	
		**North Africa & Middle East**	10 (2.7)	43 (2.3)	16 (2.1)	
		**South East Asia**	46 (12.4)a, b	73 (3.9)a	29 (3.8)b	
		**North East Asia**	24 (6.5)b	88 (4.7)c	16 (2.1)b, c	
		**South and Central Asia**	117 (31.6)a, b	318 (16.8)a	116 (15.4)b	
		**America (North & South)**	6 (1.6)	38 (2.0)	13 (1.7)	
		**Sub Saharan Africa**	9 (2.4)	32 (1.7)	11 (1.5)	
		**Unknown**	1 (0.3)	22 (1.2)	72 (9.5)	
	**English Speaking Background**	**Yes**	153 (41.4)a, b	1245 (65.8)a	509 (67.5)b	**<0.001**
		**No**	217 (58.6)a, b	646 (34.2)a	245 (32.5)b	
**O**	**Employment Status**	**Employed**	264 (71.4)	1275 (67.4)	489 (64.9)	**<0.001**

Variable			*Life!* GDM (N=370)	*Life!* program (N=2645)		*p*-value
			**2022-2025**	**2022-2025 (n=1891)**	**2018-2021 (n=754)**	
		**Home duties**	70 (18.9)a	167 (8.8)a, c	146 (19.4)c	
		**Unemployed/Retired**	14 (3.8)	88 (4.7)c	76 (10.1)c	
		**Student**	9 (2.4)a	14 (0.7)a	12 (1.6)	
		**Unknown**	13 (3.5)	347 (18.4)	31 (4.1)	
**E**	**Education Level**	**Tertiary education**	343 (92.7)a, b	1253 (66.3)a	480 (63.7)b	**<0.001**
		**No tertiary education**	17 (4.6)a, b	215 (11.4)a, c	244 (32.4)b, c	
		**Unknown**	10 (2.7)	423 (22.4)	30 (4.0)	
**S**	**Income Level**	**High**	101 (27.3)a, b	102 (5.4)a, c	108 (14.3)b, c	**<0.001**
		**Medium**	155 (41.9)	750 (39.7)c	367 (48.7)c	
		**Low**	51 (13.8)a, b	500 (26.4)a	174 (23.1)b	
		**Unknown**	63 (17.0)	539 (28.5)	105 (13.9)	
**Others**	**Current Age at Enrolment**		38.0 (35.0, 41.0)	37.0 (34.0, 43.0)	41.0 (36.0, 51.0)	**<0.001**
	**Marital Status**	**De facto/married**	337 (91.1)a, b	1385 (73.2)a, c	601 (79.7)b, c	**<0.001**
		**Divorced/separated**	14 (3.8)b	81 (4.3)c	64 (8.5)b, c	
		**Never married/widowed**	12 (3.2)	59 (3.1)c	47 (6.2)c	
		**Unknown**	7 (1.9)	366 (19.4)	42 (5.6)	
	**Do you Smoke Daily?**	**No**	363 (98.1)b	1843 (97.5)c	687 (91.1)b, c	**<0.001**
		**Yes**	7 (1.9)b	47 (2.5)c	45 (6.0)b, c	
		**Unknown**	0 (0.0)	1 (0.1)	22 (2.9)	
	**Referral Channel**	**Health Facilitator/Provider**	1 (0.3)a, b	492 (26.0)a, c	118 (15.6)b, c	**<0.001**
		**Health Professional/GP/Pharmacist**	8 (2.2)a, b	208 (11.0)a, c	230 (30.5)b, c	
		**Self**	357 (96.5)a, b	1191 (63.0)a, c	406 (53.8)b, c	
		**Unknown**	4 (1.1)	0 (0.0)	0 (0.0)	

* GDM, gestational diabetes mellitus. a indicates a significant difference between *Life!* GDM (n=370) and *Life!* (2022-2025) after Benjamini-Hochberg adjustment; b indicates a significant difference between *Life!* GDM (n=370) and *Life!* (2018-2021) after Benjamini-Hochberg adjustment; c indicates a significant difference between *Life!* (2022-2025) and *Life!* (2018-2021) after Benjamini-Hochberg adjustment.

Table S3. Program completion by participant characteristics in *Life!* GDM (2022-2025)

Variable			Not completed n=234 (63.2%)	Completed n=136 (36.7%)	Overall p-value
P	Area	Metropolitan	202 (86.3%)	116 (85.3%)	0.905
		Regional	32 (13.7%)	20 (14.7%)	
R	Country of Birth	Oceania	84 (35.9%)	58 (42.6%)	0.236
		Europe and America	15 (6.4%)	9 (6.6%)	
		Africa	15 (6.4%)	8 (5.9%)	
		South East and North East Asia	39 (16.7%)	29 (21.3%)	
		South and Central Asia	81 (34.6%)	32 (23.5%)	
	Cultural Background	Oceania	83 (35.5%)	58 (42.6%)	0.151
		Europe and America	14 (6.0%)	8 (5.9%)	
		Africa	14 (6.0%)	5 (3.7%)	
		South East and North East Asia	39 (16.7%)	31 (22.8%)	
		South and Central Asia	83 (35.5%)	34 (25.0%)	
		Unknown	1 (0.4%)	0 (0.0%)	
	English Speaking Background	Yes	90 (38.5%)	63 (46.3%)	0.170
		No	144 (61.5%)	73 (53.7%)	
O	Employment Status	Employed	164 (70.1%)	100 (73.5%)	0.483
		Home duties	44 (18.8%)	26 (19.1%)	
		Unemployed/Retired	9 (3.8%)	5 (3.7%)	
		Student	8 (3.4%)	1 (0.7%)	
		Unknown	9 (3.8%)	4 (2.9%)	
E	Education Level	Tertiary education	217 (92.7%)	126 (92.6%)	0.723
		No tertiary education	12 (5.1%)	5 (3.7%)	
		Unknown	5 (2.1%)	5 (3.7%)	
S	Income Level	High	60 (25.6%)	41 (30.1%)	0.402
		Medium	98 (41.9%)	57 (41.9%)	
		Low	36 (15.4%)	15 (11.0%)	

Variable			Not completed<br>n=234 (63.2%)	Completed<br>n=136 (36.7%)	Overall *p*-value
		**Unknown**	40 (17.1%)	23 (16.9%)	
**Others**	**Current Age at Enrolment**		37.9 (5.2)	38.5 (4.6)	0.261
	**Marital Status**	**Defacto/married**	211 (90.2%)	126 (92.6%)	0.102
		**Divorced/separated**	10 (4.3%)	4 (2.9%)	
		**Never married/widowed**	11 (4.7%)	1 (0.7%)	
		**Unknown**	2 (0.9%)	5 (3.7%)	
	**Do you Smoke Daily?**	**No**	229 (97.9%)	134 (98.5%)	1.000
		**Yes**	5 (2.1%)	2 (1.5%)	
	**Referral Channel**	**Health Facilitator/Provider**	0 (0.0%)	1 (0.7%)	0.537
		**Health Professional/GP/Pharmacist**	5 (2.1%)	3 (2.2%)	
		**Self**	226 (96.6%)	131 (96.3%)	
		**Unknown**	3 (1.3%)	1 (0.7%)	
		**No**	199 (85.0%)	121 (89.0%)	

GDM, gestational diabetes mellitus.

Table S4. Program completion by participant characteristics in *Life!* (2022-2025)

		Variable	**Not completed** **n=903 (47.8%)**	**Completed** **n=988 (52.2%)**	***p*-value**
**P**	**Area**	**Metropolitan**	717 (79.4%)	776 (78.5%)	0.688
		**Regional**	186 (20.6%)	212 (21.5%)	
	**Income Level**	**High**	51 (5.6%)	51 (5.2%)	0.173
		**Medium**	315 (34.9%)	435 (44.0%)	
		**Low**	230 (25.5%)	270 (27.3%)	
		**Unknown**	307 (34.0%)	232 (23.5%)	
**R**	**Country of Birth**	**Oceania**	541 (59.9%)	633 (64.1%)	**0.014**
		**Europe and America**	58 (6.4%)	80 (8.1%)	
		**Africa**	49 (5.4%)	32 (3.2%)	
		**South East and North East Asia**	78 (8.6%)	86 (8.7%)	
		**South and Central Asia**	172 (19.0%)	150 (15.2%)	
		**Unknown**	5 (0.6%)	7 (0.7%)	
	**Cultural Background**	**Oceania**	547 (60.6%)	620 (62.8%)	**0.024**
		**Europe and America**	61 (6.8%)	87 (8.8%)	
		**Africa**	45 (5.0%)	30 (3.0%)	
		**South East and North East Asia**	74 (8.2%)	87 (8.8%)	
		**South and Central Asia**	169 (18.7%)	149 (15.1%)	
		**Unknown**	7 (0.8%)	15 (1.5%)	
	**English Speaking Background**	**Yes**	571 (63.2%)	674 (68.2%)	**0.025**
		**No**	332 (36.8%)	314 (31.8%)	
**O**	**Employment Status**	**Employed**	565 (62.6%)	710 (71.9%)	0.391
		**Home duties**	84 (9.3%)	83 (8.4%)	
		**Unemployed/Retired**	44 (4.9%)	44 (4.5%)	
		**Student**	6 (0.7%)	8 (0.8%)	
		**Unknown**	204 (22.6%)	143 (14.5%)	
**E**	**Education Level**	**Tertiary education**	574 (63.6%)	679 (68.7%)	0.382

Variable		Not completed<br>n=903 (47.8%)	Completed<br>n=988 (52.2%)	*p*-value
	**No tertiary education**	91 (10.1%)	124 (12.6%)	
	**Unknown**	238 (26.4%)	185 (18.7%)	
**OthersCurrent Age at Enrolment**		40.4 (10.4)	40.3 (10.5)	0.876
**Marital Status**	**Defacto/married***	615 (68.1%)	770 (77.9%)	**0.003**
	**Divorced/separated***	49 (5.4%)	32 (3.2%)	
	**Never married/widowed**	34 (3.8%)	25 (2.5%)	
	**Unknown**	205 (22.7%)	161 (16.3%)	
**Do you Smoke Daily?**	**No**	877 (97.1%)	966 (97.8%)	0.368
	**Yes**	26 (2.9%)	21 (2.1%)	
	**Unknown**	0 (0.0%)	1 (0.1%)	
**Referral Channel**	**Health Facilitator/Provider***	269 (29.8%)	223 (22.6%)	**<0.001**
	**Health Professional/GP/Pharmacist***	128 (14.2%)	80 (8.1%)	
	**Self***	506 (56.0%)	685 (69.3%)	

GDM, gestational diabetes mellitus. * indicates statistically significant differences between the two programs after Benjamini-Hochberg adjustment.

Table S5. Program completion by participant characteristics in *Life!* (2018-2021)

	Variable		Not completed n=319 (42.3%)	Completed n=435 (57.7%)	*p*-value
P	**Area**	**Metropolitan**	222 (69.6%)	340 (78.2%)	**0.010**
		**Regional**	97 (30.4%)	95 (21.8%)	
	**Income Level**	**High***	56 (17.6%)	52 (12.0%)	**<0.001**
		**Medium***	119 (37.3%)	248 (57.0%)	
		**Low***	88 (27.6%)	86 (19.8%)	
		**Unknown**	56 (17.6%)	49 (11.3%)	
R	**Country of Birth**	**Oceania**	208 (65.2%)	278 (63.9%)	0.741
		**Europe and America**	29 (9.1%)	42 (9.7%)	
		**Africa**	11 (3.4%)	21 (4.8%)	
		**South East and North East Asia**	16 (5.0%)	28 (6.4%)	
		**South and Central Asia**	52 (16.3%)	63 (14.5%)	
		**Unknown**	3 (0.9%)	3 (0.7%)	
	**Cultural Background**	**Oceania**	173 (54.2%)	246 (56.6%)	0.662
		**Europe and America**	36 (11.3%)	39 (9.0%)	
		**Africa**	9 (2.8%)	18 (4.1%)	
		**South East and North East Asia**	17 (5.3%)	28 (6.4%)	
		**South and Central Asia**	50 (15.7%)	66 (15.2%)	
		**Unknown**	34 (10.7%)	38 (8.7%)	
	**English Speaking Background**	**Yes**	216 (67.7%)	293 (67.4%)	0.981
		**No**	103 (32.3%)	142 (32.6%)	
O	**Employment Status**	**Employed**	207 (64.9%)	282 (64.8%)	0.934
		**Home duties**	60 (18.8%)	86 (19.8%)	
		**Unemployed/Retired**	31 (9.7%)	45 (10.3%)	
		**Student**	6 (1.9%)	6 (1.4%)	
		**Unknown**	15 (4.7%)	16 (3.7%)	
E	**Education Level**	**Tertiary education**	210 (65.8%)	270 (62.1%)	0.399

Variable		Not completed n=319 (42.3%)	Completed n=435 (57.7%)	*p*-value
	**No tertiary education**	98 (30.7%)	146 (33.6%)	
	**Unknown**	11 (3.4%)	19 (4.4%)	
**OthersCurrent Age at Enrolment**		44.9 (11.6)	43.7 (11.3)	0.164
**Marital Status**	**Defacto/married**	251 (78.7%)	350 (80.5%)	0.312
	**Divorced/separated**	27 (8.5%)	37 (8.5%)	
	**Never married/widowed**	25 (7.8%)	22 (5.1%)	
	**Unknown**	16 (5.0%)	26 (6.0%)	
**Do you Smoke Daily?**	**No**	283 (88.7%)	404 (92.9%)	**0.020**
	**Yes**	27 (8.5%)	18 (4.1%)	
	**Unknown**	9 (2.8%)	13 (3.0%)	
**Referral Channel**	**Health Facilitator/Provider**	58 (18.2%)	60 (13.8%)	**<0.001**
	**Health Professional/GP/Pharmacist***	123 (38.6%)	107 (24.6%)	
	**Self***	138 (43.3%)	268 (61.6%)	

GDM, gestational diabetes mellitus. * indicates statistically significant differences between the two programs after Benjamini-Hochberg adjustment.

Table S6. Regression analyses of program completion by participant characteristics in *Life!* participants (2018-2021)

Variable	Category	COR (95% CI)	*p*-value	AOR (95% CI)	*p*-value
**Area**	**Metropolitan (ref)**				
	**Regional**	0.64 (0.46-0.89)	0.008	0.65 (0.43-1.00)	**0.048**
**Country of Birth**	**Oceanian (ref)**				
	**Europe and America**	1.08 (0.66-1.81)	0.756	0.78 (0.32-1.98)	0.598
	**Africa**	1.43 (0.69-3.13)	0.352	0.90 (0.28-3.05)	0.860
	**South East and North East Asia**	1.31 (0.70-2.53)	0.409	1.16 (0.33-4.24)	0.816
	**South and Central Asia**	0.91 (0.60-1.37)	0.638	0.50 (0.16-1.51)	0.213
**English Speaking Background**	**Yes (ref)**				
	**No**	1.02 (0.75-1.39)	0.918	1.39 (0.50-3.77)	0.519
**Education Level**	**Tertiary education (ref)**				
	**No tertiary education**	1.16 (0.85-1.59)	0.356	1.24 (0.84-1.85)	0.280
**Employment Status**	**Employed (ref)**				
	**Home duties**	1.05 (0.72-1.54)	0.791	1.51 (0.94-2.47)	0.097
	**Unemployed/Retired**	1.07 (0.65-1.75)	0.800	1.11 (0.53-2.37)	0.777
	**Student**	0.73 (0.23-2.38)	0.597	2.61 (0.63-13.27)	0.203
**Income Level**	**High (ref)**				
	**Medium**	2.24 (1.45-3.48)	<0.001	1.70 (1.03-2.82)	**0.039**
	**Low**	1.05 (0.65-1.70)	0.835	0.75 (0.40-1.41)	0.377

Variable	Category	COR (95% CI)	*p*-value	AOR (95% CI)	*p*-value
**Marital Status**	**De facto/married (ref)**				
	**Single (divorced, separated, widowed, or never married)**	0.81 (0.54-1.22)	0.320	1.35 (0.79-2.33)	0.273
**Current age at Enrolment**		0.99 (0.98-1.00)	0.163	1.01 (0.98-1.03)	0.642
**Do you Smoke Daily?**	**No (ref)**				
	**Yes**	0.47 (0.25-0.86)	0.015	0.67 (0.30-1.48)	0.323
**Referral Channel**	**Healthcare professional (ref)**				
	**Self**	2.10 (1.57-2.83)	<0.001	1.90 (1.29-2.81)	**0.001**

GDM, gestational diabetes mellitus; COR, crude odds ratio; AOR, adjusted odds ratio. Hosmer-Lemeshow goodness-of-fit: χ^2=7.96, df=8, p=0.437

Table S7. Univariable regression analyses of program completion by participant characteristics in *Life!* GDM (2022-2025).

Variable	Category	COR (95% CI)	p-value
**Area**	**Metropolitan (ref)**		
	**Regional**	1.09 (0.59-1.98)	0.783
**Country of Birth**	**Oceanian (ref)**		
	**Europe and America**	0.87 (0.34-2.09)	0.758
	**Africa**	0.77 (0.29-1.90)	0.583
	**South East and North East Asia**	1.08 (0.60-1.93)	0.804
	**South and Central Asia**	**0.57 (0.33-0.97)**	**0.038**
**English Speaking Background**	**Yes (ref)**		
	**No**	0.72 (0.47-1.11)	0.139
**Education Level**	**Tertiary education (ref)**		
	**No tertiary education**	0.72 (0.22-1.98)	0.542
**Employment Status**	**Employed (ref)**		
	**Home duties**	0.97 (0.56-1.66)	0.910
	**Unemployed/Retired**	0.91 (0.27-2.71)	0.871
	**Student**	0.21 (0.01-1.14)	0.138
**Income Level**	**High (ref)**		
	**Medium**	0.85 (0.51-1.43)	0.539
	**Low**	0.61 (0.29-1.24)	0.179
**Marital Status**	**De facto/married (ref)**		
	**Single (divorced, separated, widowed, or never married)**	0.40 (0.13-1.01)	0.072

Variable	Category	COR (95% CI)	p-value
**Current Age at Enrolment**		1.02 (0.98-1.07)	0.274
**Do you Smoke Daily?**	**No (ref)**		
	**Yes**	0.68 (0.10-3.22)	0.652
**Referral Channel**	**Healthcare professional (ref)**		
	**Self**	0.72 (0.19-2.97)	0.636

GDM, gestational diabetes mellitus; COR, crude odds ratio.

Table S8. Univariable regression analyses of program completion by participant characteristics in *Life!* participants with a history of GDM (2022-2025).

Variable	Category	COR (95% CI)	p-value
**Area**	Metropolitan (ref)		
	Regional	1.05 (0.84-1.31)	0.647
**Country of Birth**	Oceanian (ref)		
	Europe and America	1.18 (0.83-1.69)	0.366
	Africa	**0.56 (0.35-0.88)**	**0.013**
	South East and North East Asia	0.94 (0.68-1.31)	0.722
	South and Central Asia	**0.75 (0.58-0.95)**	**0.020**
**English Speaking Background**	Yes (ref)		
	No	**0.80 (0.66-0.97)**	**0.023**
**Education Level**	Tertiary education (ref)		
	No tertiary education	1.15 (0.86-1.55)	0.343
**Employment Status**	Employed (ref)		
	Home duties	0.79 (0.57-1.09)	0.144
	Unemployed/Retired	0.80 (0.52-1.23)	0.300
	Student	1.06 (0.37-3.24)	0.913
**Income Level**	High (ref)		
	Medium	1.38 (0.91-2.09)	0.127
	Low	1.17 (0.77-1.80)	0.461
**Marital Status**	De facto/married (ref)		
	Single (divorced, separated, widowed, or never married)	**0.55 (0.38-0.78)**	**<0.001**

Variable	Category	COR (95% CI)	p-value
**Current age at Enrolment**		1.00 (0.99-1.01)	0.876
**Do you Smoke Daily?**	No (ref)		
	Yes	0.73 (0.41-1.31)	0.296
**Referral Channel**	Healthcare professional (ref)		
	Self	**1.77 (1.47-2.14)**	**<0.001**

GDM, gestational diabetes mellitus; COR, crude odds ratio.

Table S9. Multivariable regression analyses of program completion by participant characteristics in Life! GDM participants with a history of GDM (2022-2025).

Variable	Category	AOR	Std. Err.	*z*	*p*	95% CI
**Area**	Metropolitan (ref)					
	Regional	0.96	0.36	-0.11	0.916	0.46-2.02
**Country of Birth**	Oceanian (ref)					
	Europe & Americas	0.94	0.64	-0.09	0.928	0.25-3.58
	Africa & Middle East	2.38	2.23	0.92	0.356	0.38-14.95
	East Asia	3.99	3.67	1.50	0.133	0.65-24.26
	South & Central Asia	1.42	1.29	0.39	0.697	0.24-8.38
**English-speaking background**	Yes (ref)					
	No	0.30	0.26	-1.40	0.162	0.06-1.62
**Education level**	Tertiary education (ref)					
	Not Tertiary	0.82	0.54	-0.30	0.761	0.22-3.01
**Employment status**	Employed (ref)					
	Home Duties	1.79	0.67	1.54	0.125	0.85-3.74
	Retired/Unemployed	4.26	3.99	1.55	0.122	0.68-26.66
	Student	0.49	0.56	-0.62	0.536	0.05-4.58
**Income level**	High (ref)					
	Moderate	0.89	0.26	-0.41	0.685	0.51-1.56

	Low	0.75	0.34	-0.64	0.523	0.31-1.80
**Marital status**	De facto/married (ref)					
	Single (divorced, separated, widowed, or never married)	0.06	0.06	-2.68	**0.007**	0.01-0.47
**Current age**	(continuous)	1.03	0.03	1.21	0.225	0.98-1.09
**Smoker**	No (ref)					
	Yes	0.62	0.57	-0.52	0.602	0.10-3.71
**Referral channel**	Healthcare professional (ref)					
	Self	0.69	0.68	-0.37	0.708	0.10-4.79
**_cons**		0.33	0.48	-0.76	0.445	0.02-5.59

Table S10. Multivariable regression analyses of program completion by participant characteristics in Life! participants with a history of GDM (2022-2025).

Variable	Category	AOR	Std. Err.	*z*	*p*	95% CI
**Area**	Metropolitan (ref)					
	Regional	1.11	0.16	0.68	0.497	0.83-1.48
**Country of Birth**	Oceanian (ref)					
	Europe & Americas	1.23	0.39	0.64	0.520	0.66-2.28
	Africa & Middle East	0.75	0.33	-0.67	0.502	0.32-1.76
	East Asia	1.35	0.58	0.70	0.482	0.58-3.13
	South & Central Asia	1.02	0.41	0.06	0.955	0.46-2.26
**English-speaking background**	Yes (ref)					
	No	0.79	0.30	-0.62	0.535	0.38-1.65
**Education level**	Tertiary education (ref)					
	Not Tertiary	1.27	0.21	1.39	0.164	0.91-1.76
**Employment status**	Employed (ref)					
	Home Duties	0.61	0.13	-2.38	**0.017**	0.41-0.92
	Retired/Unemployed	0.58	0.17	-1.82	0.069	0.33-1.04
	Student	1.35	0.80	0.51	0.610	0.43-4.28
**Income level**	High (ref)					
	Moderate	1.38	0.31	1.40	0.160	0.88-2.15
	Low	1.29	0.31	1.08	0.278	0.81-2.06
**Marital status**	De facto/married (ref)					

	Single (divorced, separated, widowed, or never married)	0.53	0.12	-2.86	**0.004**	0.34-0.82
**Current age**	(continuous)	1.01	0.01	1.55	0.122	1.00-1.03
**Smoker**	No (ref)					
	Yes	0.61	0.23	-1.32	0.188	0.29-1.27
**Referral channel**	Healthcare professional (ref)					
	Self	1.35	0.22	1.84	0.066	0.98-1.86
**_cons**		0.54	0.23	-1.47	0.141	0.24-1.23

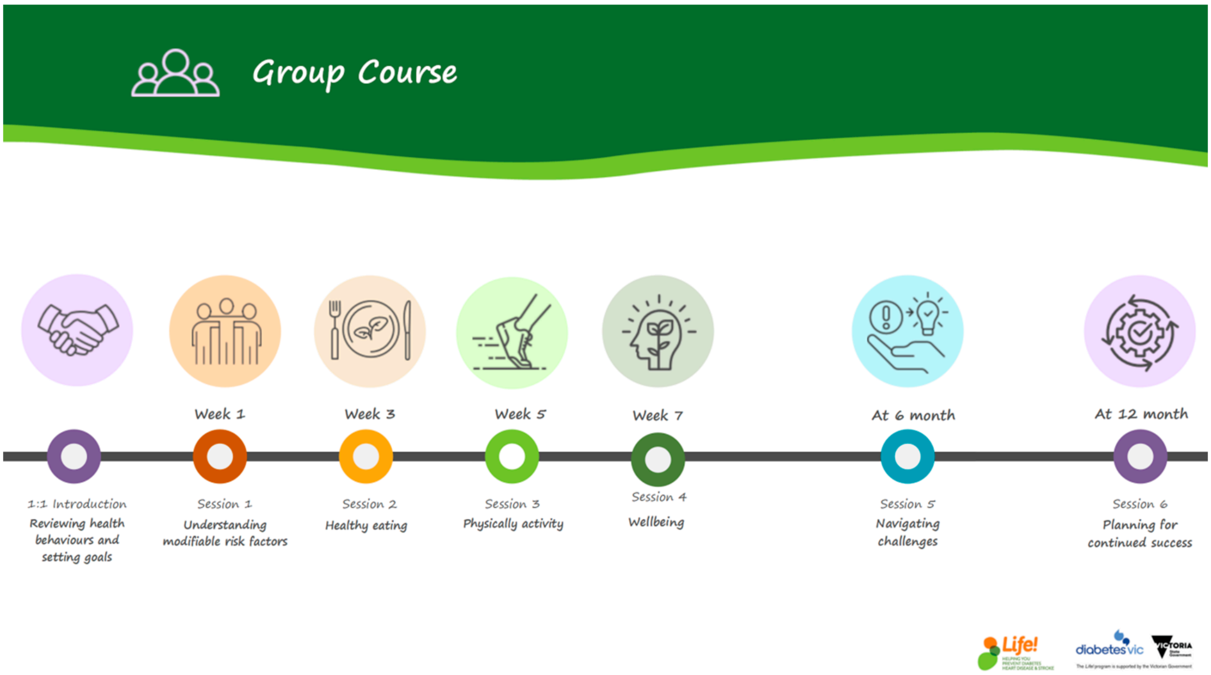

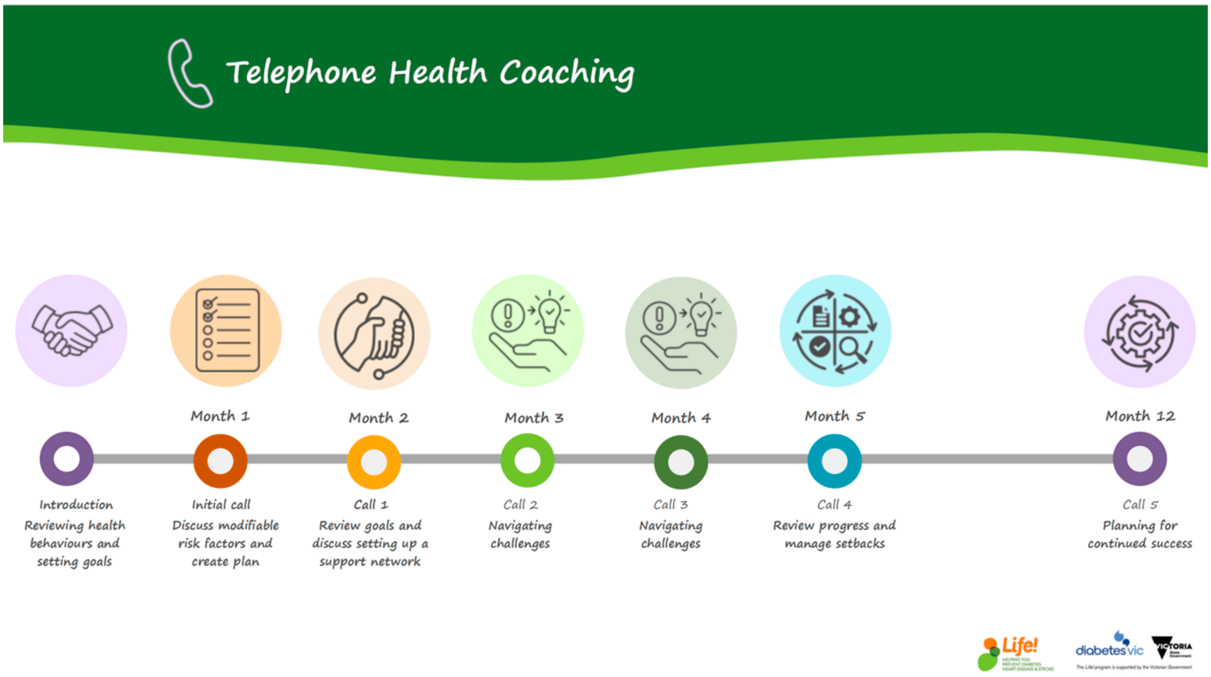

Figures S1. Program design.
